# Radiopathologic Characteristics of Invasive Mammary Carcinoma With Medullary Features: A Correlative Study

**DOI:** 10.7759/cureus.92158

**Published:** 2025-09-12

**Authors:** Sanjanika S, Pavithra V, Preetha Nethaji, Bhawna Dev

**Affiliations:** 1 Pathology, Sri Ramachandra Institute of Higher Education and Research, Chennai, IND; 2 Radiology and Imaging Sciences, Anderson Diagnostics and Labs, Chennai, IND; 3 Radiology and Imaging Sciences, Sri Ramachandra Institute of Higher Education and Research, Chennai, IND; 4 Radiology, Sri Ramachandra Institute of Higher Education and Research, Chennai, IND

**Keywords:** breast, histopathology, imaging, mammography, medullary carcinoma breast (mcb), ultrasound

## Abstract

Introduction

Invasive mammary carcinoma with medullary features represents an uncommon subtype of breast cancer. Despite their high-grade histological appearances, they have a favourable prognosis. This study aims to correlate its radiologic and histopathologic characteristics. A comprehensive understanding of the radiopathologic profile is essential for enhancing the diagnosis precision and guiding patient treatment, particularly because of its typically benign imaging findings, which may result in misinterpretation and underdiagnosis.

Materials and methods

A retrospective observational study was conducted by reviewing cases of histologically confirmed invasive mammary carcinoma with medullary features and triple-negative basal-like carcinoma that met the WHO criteria over five years (2020-2025) at the Sri Ramachandra Institute of Higher Education and Research, Chennai, India. We reviewed preoperative mammographic and ultrasound features and compared them with histopathological findings. Descriptive statistics summarise the prevalence of each feature.

Results

We included a total of 45 patients (age range: 25-76 years) with a confirmed diagnosis of medullary carcinoma. On mammograms, 30 (90.9%) had a detectable mass, of which 16 (53.3%) presented as an irregular shape with circumscribed margins and equal density. Rarely were calcifications identified, and they were present only in three (10%) cases. Sonographic examination revealed 29 (83%) cases as hypoechoic; 26 (74%) irregular masses, with microlobulated margins in 18 (51.4%) cases; and posterior acoustic enhancement in 32 (91%) cases. Eighteen (51%) of the masses showed minimal internal vascularity. Histopathology confirmed that 42 (93%) of the cases showed a syncytial growth pattern, with 39 (86%) having a high nuclear grade and 44 (97.8%) showing no glandular or tubular elements. There were prominent lymphoplasmacytic infiltrates in 100% of cases, and they were all triple-negative immunophenotypes.

Conclusion

Medullary carcinoma can present similarly to benign lesions in imaging studies, exhibiting characteristics like circumscribed morphology and posterior acoustic enhancement. Despite these similarities, imaging techniques cannot definitively differentiate medullary carcinoma from other types of breast lesions, making it essential to conduct a biopsy and obtain histopathological confirmation for a conclusive, timely, and accurate diagnosis.

## Introduction

Medullary carcinoma of the breast (MCB) is a rare subtype of invasive ductal carcinoma, comprising under 5% of breast cancers. Specific histologic criteria (WHO criteria) [1] define it as a well-circumscribed mass composed of sheets of tumour cells (syncytial pattern) with high nuclear pleomorphism and dense lymphoplasmacytic infiltrates [2]. Outlined by Foote and Stewart in 1946, MCB has specific features that distinguish it from the more prevalent invasive ductal carcinoma of no special type (IMC-NST) [3]. Unlike most invasive carcinomas, they exhibit a favourable prognosis, despite their high-grade cytology [4,5].

Paradoxically, medullary carcinoma is associated with a more favourable prognosis than high-grade IMC-NST, particularly in terms of nodal involvement and long-term survival [6,7]. Despite frequently presenting as a high-grade tumour with significant nuclear pleomorphisms and high mitotic activity, medullary carcinoma is often associated with a strong host immune response [8]. MCB has a stronger association with BRCA1 mutations [9], and often it presents as a triple-negative basal-like immunophenotype (oestrogen-receptor-negative, progesterone-receptor-negative, receptor-negative, and HER2-negative) [10,11].

Clinically and radiologically, medullary carcinomas often behave in a deceptively benign fashion. On imaging, they commonly present as round or oval-shaped, circumscribed, non-calcified masses with lobulated margins. Similarly, on ultrasound, they frequently exhibit a hypoechoic mass with posterior acoustic enhancement [12]. These features overlap with benign lesions such as fibroadenomas or any cysts [13]. Because the imaging features can mimic benign entities, it is important to maintain a high level of suspicion and a comprehensive understanding of their appearance, especially when dealing with a palpable mass.

Although several studies have independently described the imaging and pathological characteristics of medullary cancer, a thorough retrospective analysis that correlates these multimodality findings within a single cohort, emphasising their diagnostic significance, is still essential for clinical practice. This study aims to provide a detailed correlative description of the mammographic, sonographic, and histopathological features of a biopsy-proven MCB, thereby improving our diagnostic expertise for this specific breast carcinoma subtype.

## Materials and methods

Study design and population

This was a single-institution, retrospective observational study conducted at Sri Ramachandra Institute of Higher Education and Research, Chennai, India. The study obtained approval from our institutional review board, which exempted the requirement for informed patient consent for the review of medical records and radiological images.

We retrospectively surveyed the electronic medical records and breast imaging databases (Picture Archiving and Communication System, PACS) to identify all consecutive female patients diagnosed with medullary carcinoma at our institution from January 2021 to May 2025. Furthermore, cases were identified using searches of the pathological database using ICD-O codes for breast carcinoma and specific histological terms, including “medullary carcinoma” and “invasive carcinoma with medullary features”.

A total of 45 cases were retrieved from the pathology department database. Four of these cases were referred from outside institutions, and among those, 17 were histologically proven to have invasive ductal carcinoma with medullary features diagnosed at our institution. The remaining 24 cases displayed a triple-negative basal-like immunophenotype and had histological characteristics like MCB (according to WHO criteria) [1].

The Inclusion criteria are patients with a definitive histopathological diagnosis of medullary carcinoma established on a core needle biopsy or surgical specimen and available preoperative mammographic and ultrasonographic imaging, with complete clinical and pathological records.

The exclusion criteria are patients having insufficient or unavailable imaging studies and patients who underwent neoadjuvant chemotherapy/radiotherapy before a conclusive diagnosis.

Data collection

Clinical and histological data, including age at diagnosis, presenting symptoms, family history, lesion size, and lymphovascular invasion, were extracted from medical records. Two independent radiologists, who specialised in breast imaging, reviewed the imaging reports (mammography and USG) without knowing the final pathological diagnosis. A breast pathologist reviewed the pathological reports, which comprised core needle biopsy and surgical excision specimens, and they were finally analysed to confirm the diagnosis.

Imaging analysis

Mammographic Examination

Mammography was performed at our institution with the usual two routine positions (craniocaudal and mediolateral) and tomosynthesis of both breasts using a direct conversion flat panel detector made of amorphous selenium with a pixel size of 50 μm (The Fujifilm Amulet Innovality, FDR MS-3500). A few of the referred cases also underwent supplementary mammograms at our institution as part of their referral process. We reviewed these mammographic images to assess the morphology of the mass (oval, round, irregular, or lobulated), looking for any asymmetry, architectural distortion, or calcification. Along with mass characteristics like margins (circumscribed, angular, spiculated, microlobulated) and density (high, isodense, low), any additional features were also reviewed.

Imaging features were systematically evaluated and documented in accordance with the BI-RADS (Breast Imaging Reporting and Data System) lexicon (5th edition) [14].

Sonographic Examination

The ultrasound images of each patient were obtained using a high-frequency linear transducer (Samsung V7 ultrasound). The images were reviewed retrospectively and assessed for mass shape (oval, round, lobulated, irregular), margins (circumscribed, microlobulated, indistinct, spiculated), echogenicity (hypoechoic, isoechoic, hyperechoic), internal echo pattern (homogeneous, heterogeneous, anechoic cystic spaces), and posterior acoustic features (enhancement, shadowing, no change) and any other additional features. We examined the sonographic images and data during the same evaluation session that followed the mammograms.

Histopathological analysis

All histopathology reports and representative H&E slides (when available for re-review) were independently assessed by a specialised breast pathologist, who was blinded to the imaging results. The diagnosis of MCB was confirmed according to the standard criteria established by the WHO classification of tumours of the breast, 5th edition (consistent with the Ridolfi criteria), which generally encompasses [15]: a syncytial growth involving at least 75% of the tumour [5], presence of a prominent lymphoplasmacytic infiltration, absence of glandular or tubular differentiation, high nuclear grade with frequent mitosis, and microscopically circumscribed pushing borders [4].

Further pathological data were gathered, which included the histological grade (Nottingham Histologic Grade), mitotic count, presence of necrosis, and the immunohistochemical (IHC) status for oestrogen, progesterone, and HER-2/neu receptors. We classified the tumours with receptor negativity as triple-negative immunophenotypes.

Statistical analysis

The frequency distribution of imaging and histological features, as well as patient demographics, was summarised using descriptive statistics. Categorical variables were presented as frequencies and percentages. When applicable, medians with interquartile ranges (IQR) or means ± standard deviations (SD) were used to express any continuous variables. Statistical analyses were done in IBM SPSS Statistics for Windows, trial version 27.0 (IBM Corp., Armonk, NY).

## Results

Patient demographics and clinical presentation

A total of 45 patients with histopathologically confirmed MCB were identified and included in the study cohort. The mean age at the final diagnosis was 53.3 years (range: 25-76 years). Most of the patients presented with a palpable lump in 38 cases (84.4%). Other less common presentations included abnormal screening mammograms in four patients (8.9%) and breast pain in three patients (6.7%). Of all the cases, 43 patients (95.6%) had solitary lesions. Out of the total cases, only two patients (4.4%) presented with multiple lesions. Three (6.7%) out of the 45 cases had a positive family history of breast cancer.

Mammographic correlation

Out of the 45 cases, 33 (73.3%) underwent mammographic analysis at our institution, and 30 of these cases (90.9%) had a detectable mass on the mammogram. Three cases (9.1%) were either excessively large or undetectable by mammography. The mean lesion size measured was 3.6 cm.

**Figure 1:**
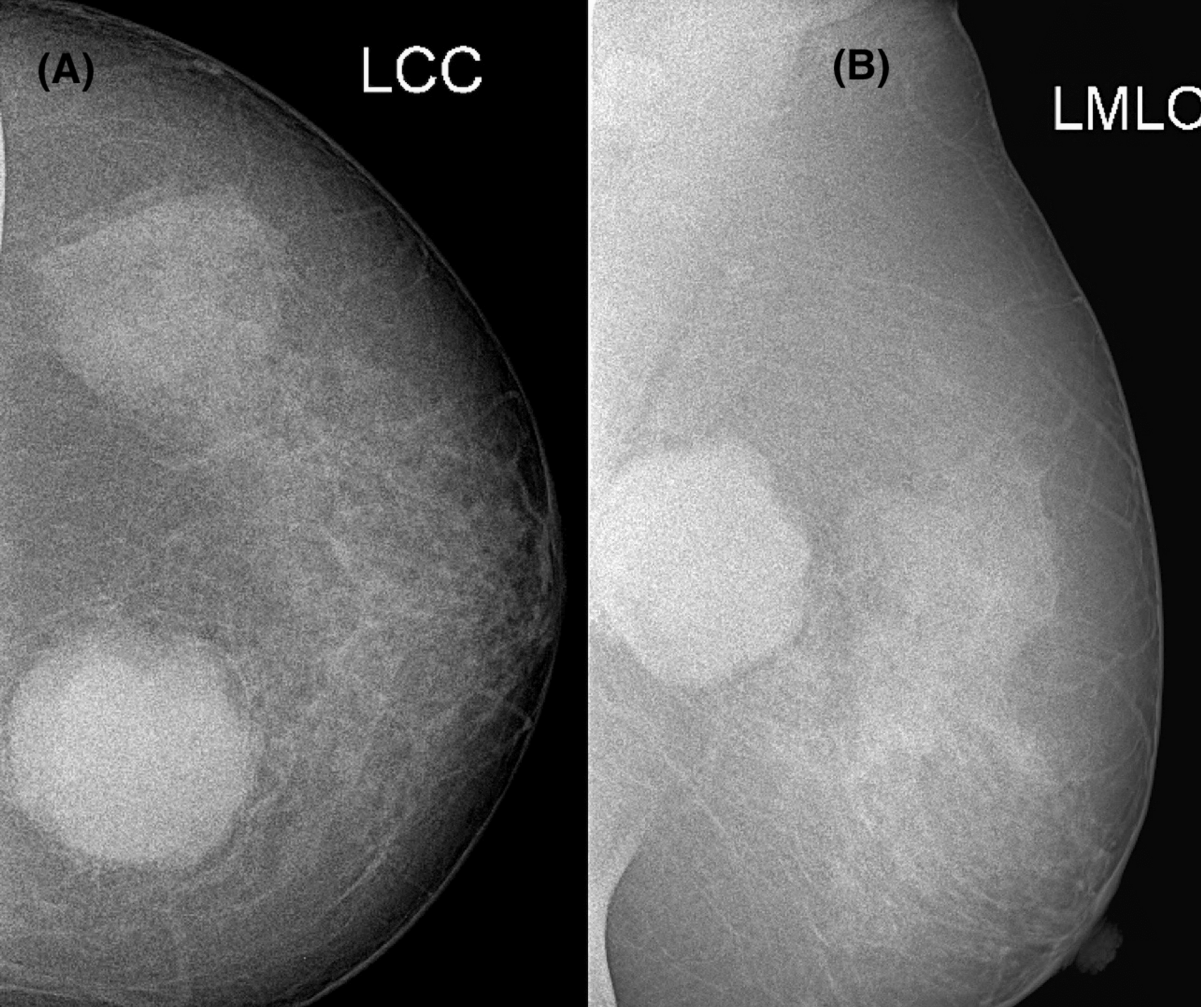
Standard craniocaudal (A) and mediolateral oblique (B) views of a mammogram of a 45-year-old female, showing a circumscribed, irregular, high-density mass in the left inner central quadrant, in the retroglandular space.

**Figure 2:**
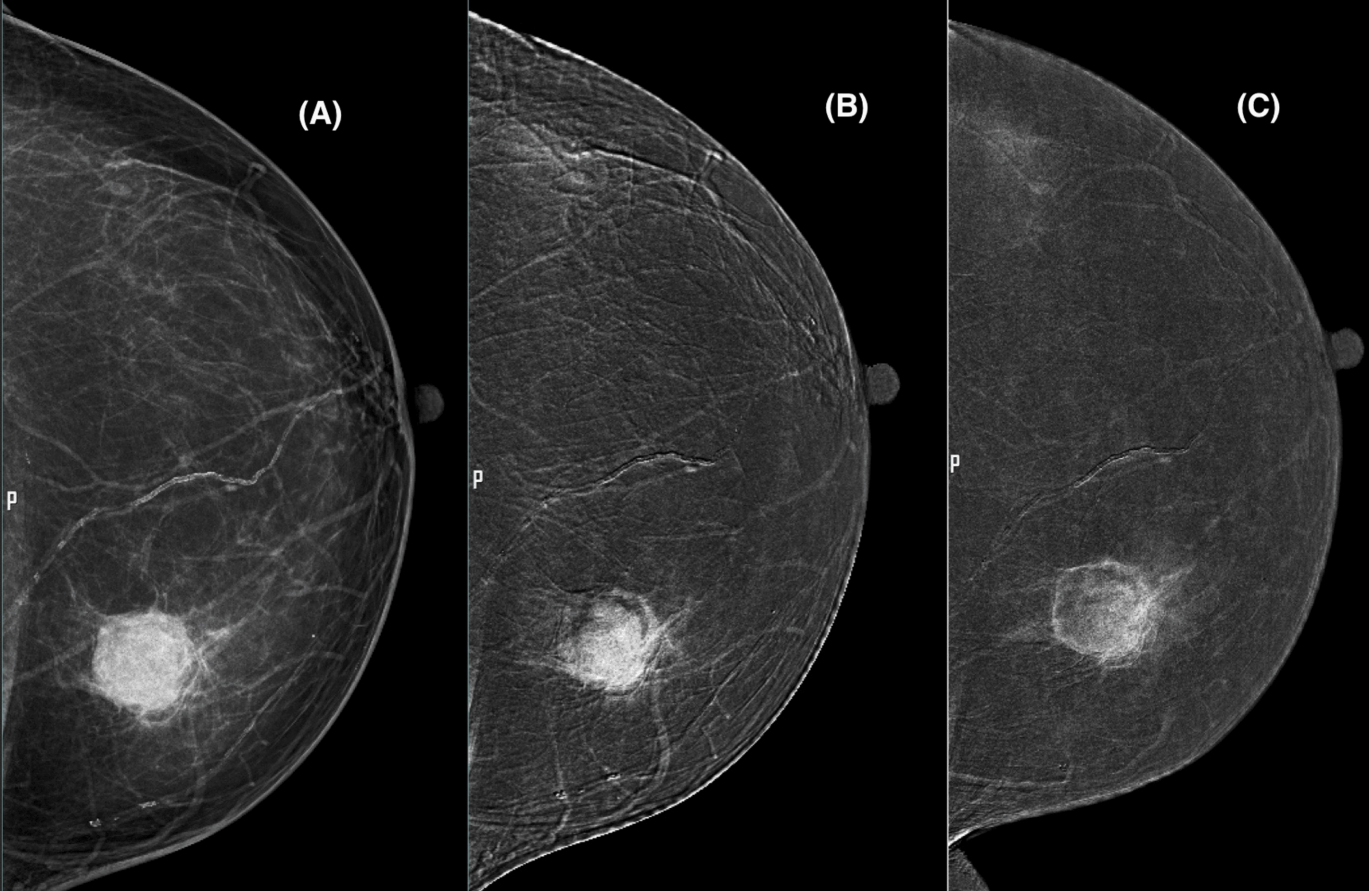
A digital mammogram in the craniocaudal view of the left breast demonstrates a circumscribed round high-density mass (A) with surrounding architectural distortion (B). Contrast-enhanced digital mammogram (recombined image) shows early intense enhancement (B) and washout (C) in the delayed image.

**Figure 3:**
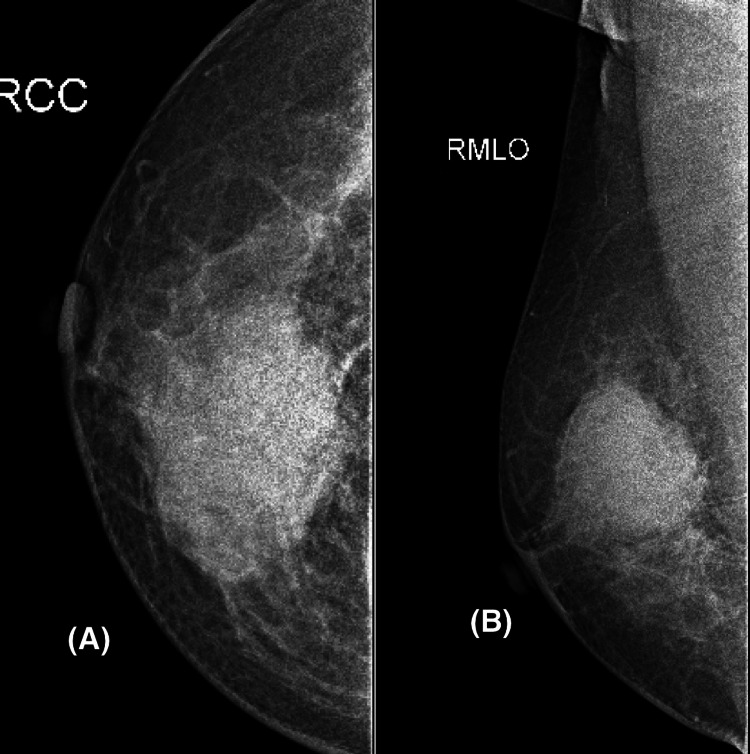
An irregular mass with partially obscured margins is seen in the mammogram (A and B) of the right breast.

**Figure 4:**
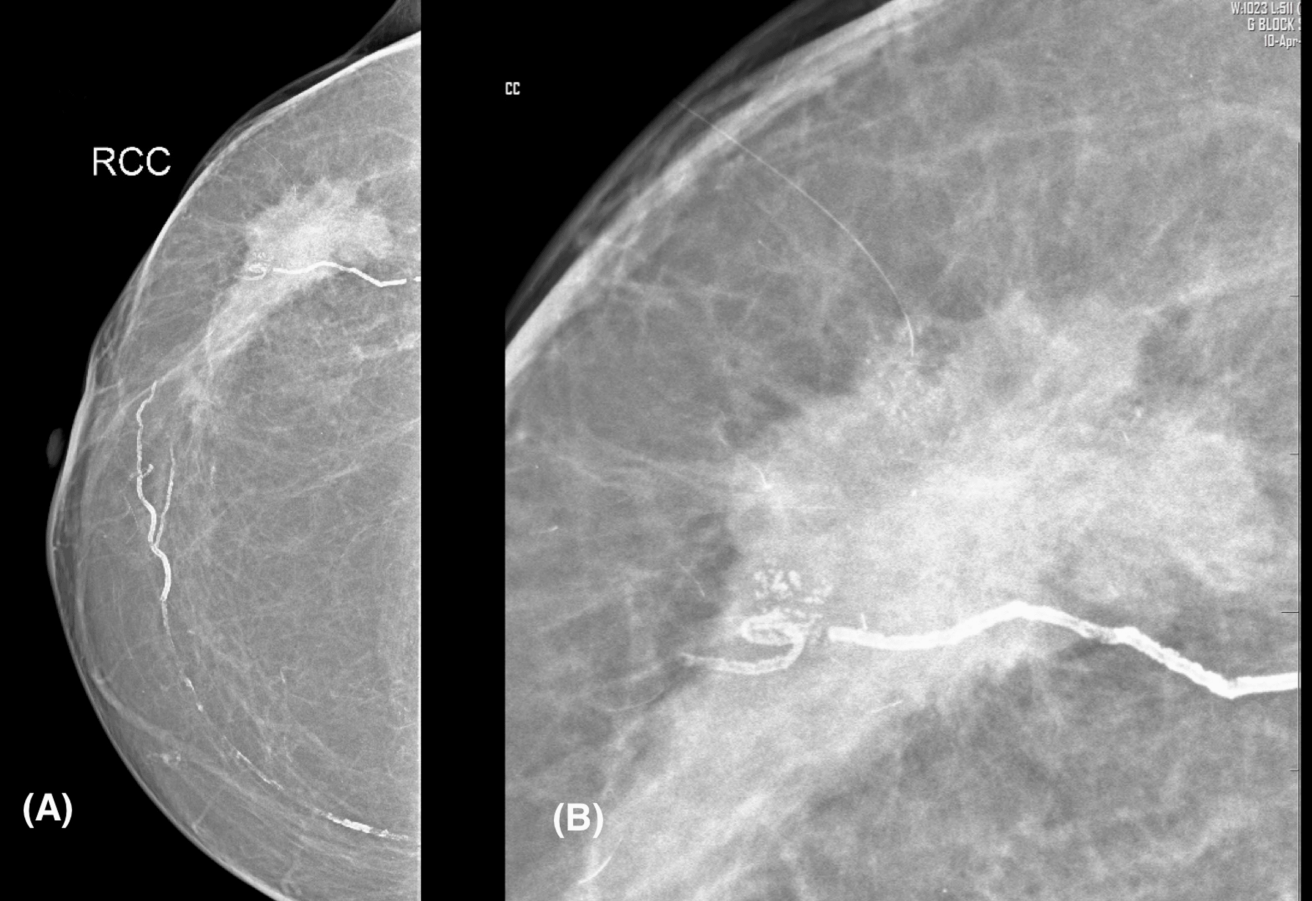
(A) The craniocaudal view of the right breast shows a high-density spiculated mass, which contains (B) grouped coarse heterogeneous calcifications that are visible in magnified images.

Of the tumours, the dominant shape was irregular in 16 cases (53.3%) (Figure 1), round in eight (26.7%) (Figure 2), and oval in six (20%) cases. The most common mammographic presentation was a mass with well-circumscribed margins, as observed in 16 (53.3%) of cases (Figure 1). Five cases (16.7%) had partially obscured margins (Figure 3), and four cases (13.3%) had microlobulated margins, while a smaller proportion, two masses (6.7%), had irregular margins. Significantly, only three cases (10%) exhibited spiculated margins (Figure 4), while two cases (6.7%) displayed irregular margins, which are characteristics highly indicative of malignancy, as noted in other breast malignancies. The density of the masses was typically isodense to the surrounding glandular tissue in 16 (53.3%) of cases and highly dense in 14 (46.7%) of cases (Figure 4). Hypodense masses were infrequently detected. Calcifications were notably uncommon in most of the tumours; they were present only as coarse, benign-appearing calcifications in three cases (10%) (Figure 4), in contrast to the fine, pleomorphic calcifications that are commonly associated with high-grade ductal carcinoma. The results are tabulated in Table 1.

**Table 1:** Summary of mammographic findings of medullary carcinoma of the breast (N = 45)

Mammographic features (n = 33)	No. of tumours (%)
Detectable mass	30 (90.9%)
Shape	
Irregular	16 (53.3%)
Oval	6 (20%)
Round	8 (26.7%)
Margins	
Well-circumscribed	16 (53.3%)
Spiculated	3 (10%)
Microlobulated	4 (13.3%)
Partially obscured	5 (16.7%)
Irregular	2 (6.7%)
Density	
Equal	16 (53.3%)
High	14 (46.7%)
Calcification	3 (10%)

 Based on the overall mammographic assessment, the lesions were categorised as BIRADS 4A in four cases (13.3%), BIRADS 4B in eight cases (26.7%), BIRADS 4C in seven cases (23.3%), and BIRADS 5 in 14 cases (36.7%).

Sonographic correlation

**Figure 5:**
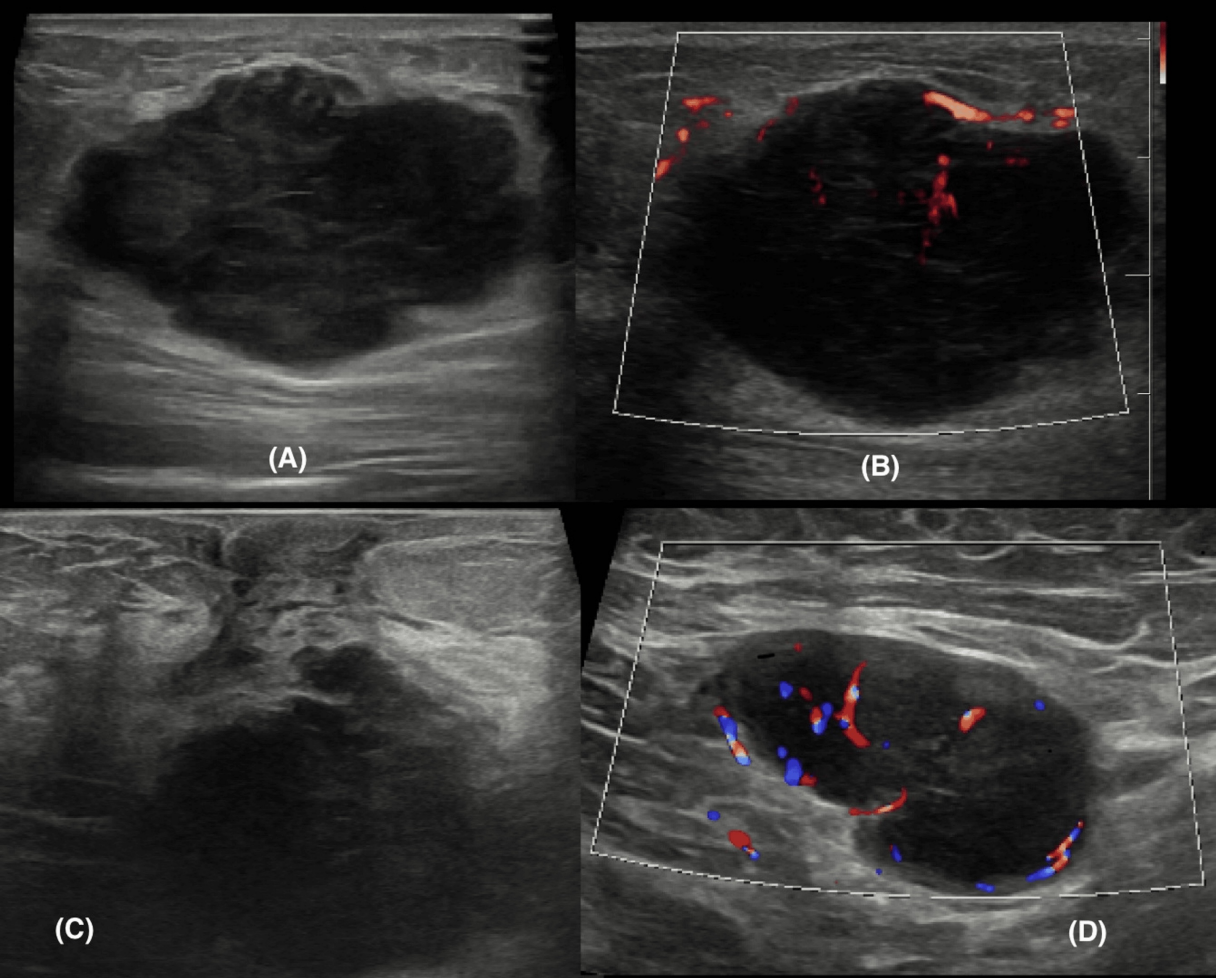
Ultrasound (A) shows a circumscribed hypoechoic irregular mass with internal vascularity on Doppler (B). An abnormal axillary lymph node with cortical thickening and increased vascularity is also noted.

**Figure 6:**
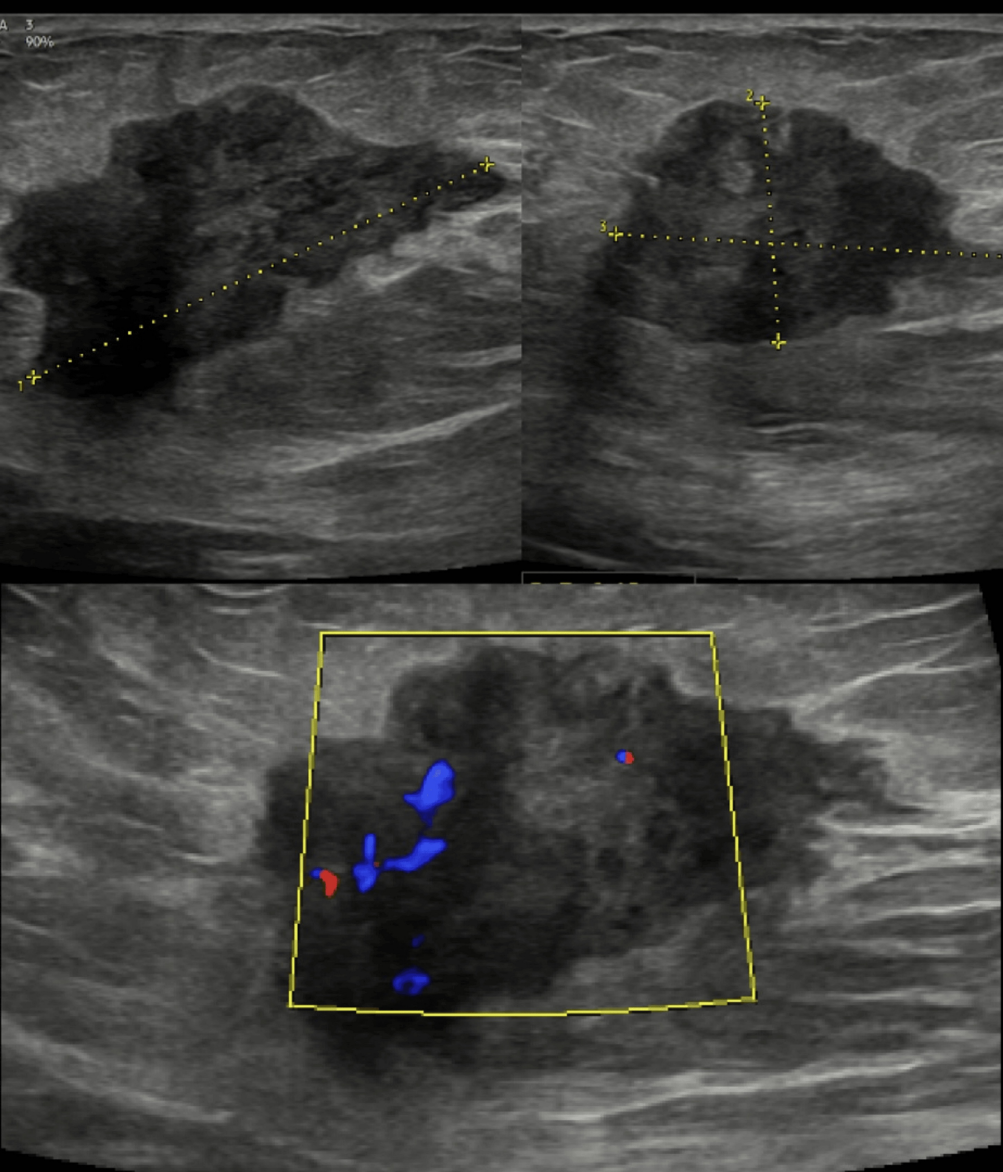
Ultrasound shows a spiculated hypoechoic mass with internal vascularity and surrounding architectural distortion.

**Figure 7:**
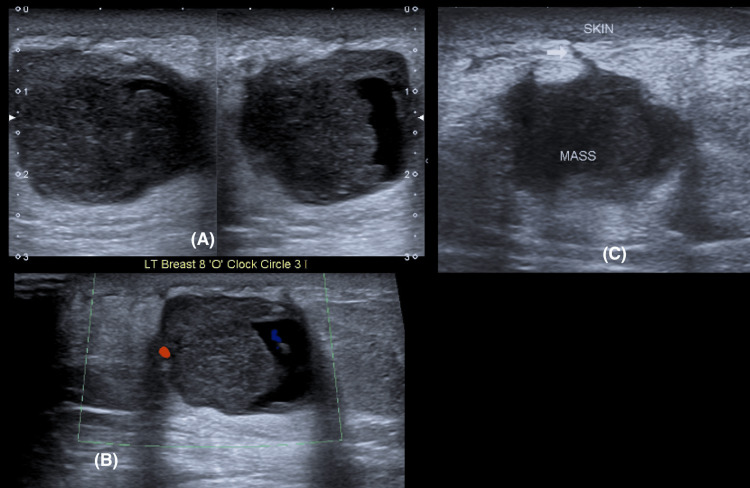
Ultrasound shows a round predominantly solid mass with crescenting cystic spaces (A) and marked posterior acoustic enhancement (B). Linear extension to the skin is also noted (C).

Breast ultrasonography images were available for review in 35 (77.8%) patients. The mean lesion size on sonography was 3.2 cm. The sonographic appearance was consistently a hypoechoic mass in 29 (83%) of cases (Figure 5), followed by heteroechoic in six (17%) cases. The predominant shapes were irregular in 26 (74%) cases (Figure 5), oval in five (14%), and round in four (11%) cases. Margins were frequently microlobulated in 18 cases (51.4%) and circumscribed in 12 cases (34.3%) (Figure 5). Angular margins were present in four cases (11.4%), and spiculated margins were rare, seen in only one case (2.9%) (Figure 6). A striking and highly prevalent finding of this lesion was posterior acoustic enhancement, which was observed in 32 (91%) cases (Figures 5, 7). On elastography, these lesions were soft in 28 (80%) cases, while some were hard in seven (20%) (Figure 6). Colour Doppler imaging revealed internal vascularity, which was 1+ (minimal) in 18 cases (51%), 2+ (moderate) in 10 (29%) of the masses, and 3+ (marked) in seven (20%) cases (Figures 5, 6). Additional features like cystic spaces were seen in five (14%) masses; three (8.5%) lesions were solid and cystic in nature (Figure 7). Three lesions (8.5%) extended to the skin (Figure 7), while four (11%) masses extended to the pectoralis muscle. The results are tabulated in Table 2.

**Table 2:** Summary of sonographic findings of medullary carcinoma of the breast (N = 45)

Sonographic features (n = 35)	No. of tumours (%)
Echogenicity	
Heteroechoic	6 (17%)
Hypoechoic	29 (83%)
Shape	
Irregular	26 (74%)
Oval	5 (14%)
Round	4 (11%)
Margins	
Microlobulated	18 (51.4%)
Circumscribed	12 (34.3%)
Angular	4 (11.4%)
Spiculated	1 (2.9%)
Posterior acoustic enhancement	32 (91%)
Elastography	
Soft	28 (80%)
Hard	7 (20%)
Internal Vascularity	
1+ (minimal)	18 (51%)
2+ (moderate)	10 (29%)
3+ (marked)	7 (20%)
Additional features	
Cystic spaces	5 (14%)
Solid and cystic	3 (8.5%)
Extension to the skin	3 (8.5%)
Extension of the pectoralis muscle	4 (11%)

Histopathological correlation

**Figure 8:**
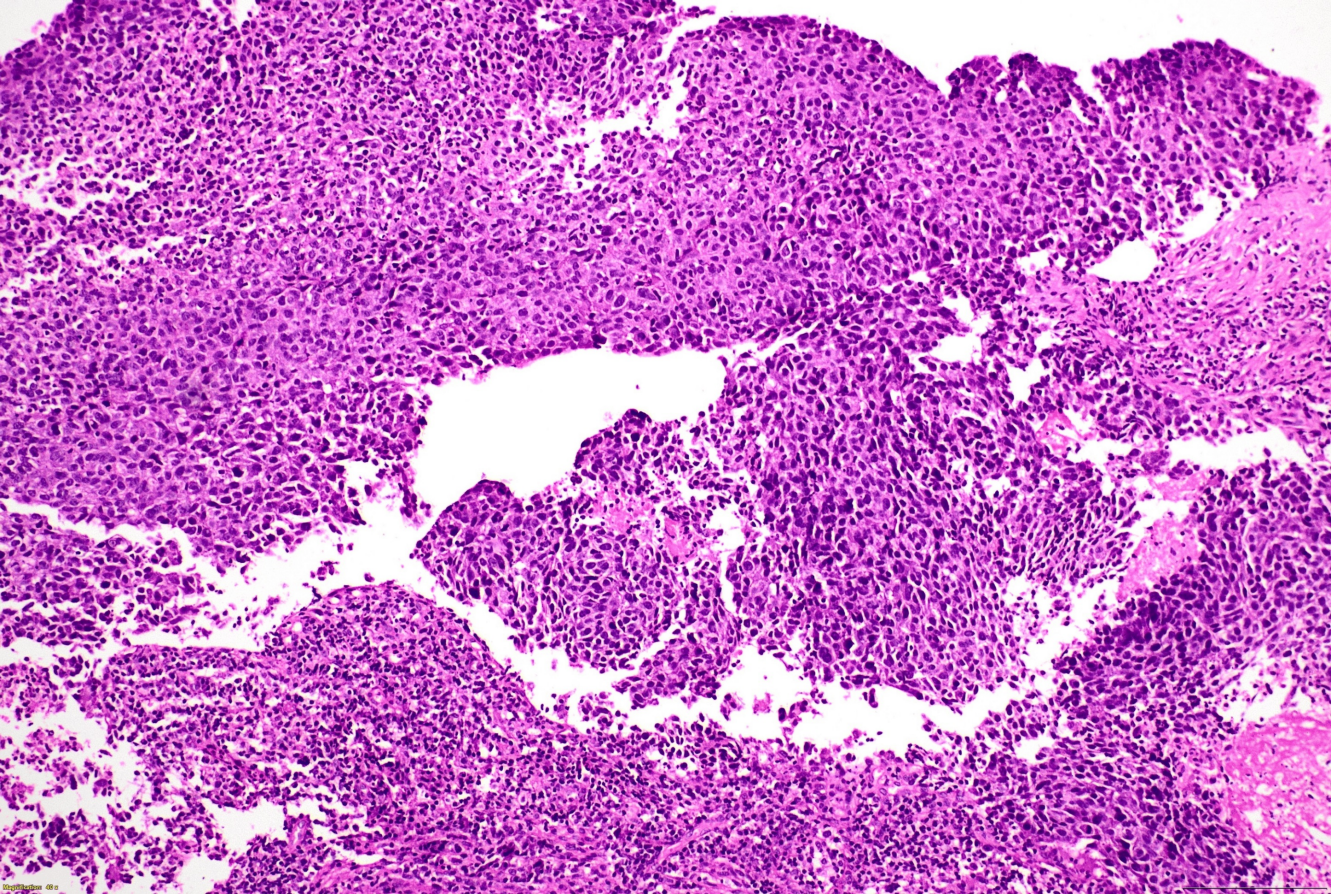
Syncytial nests of poorly differentiated tumour cells, as seen at 100x magnification.

**Figure 9:**
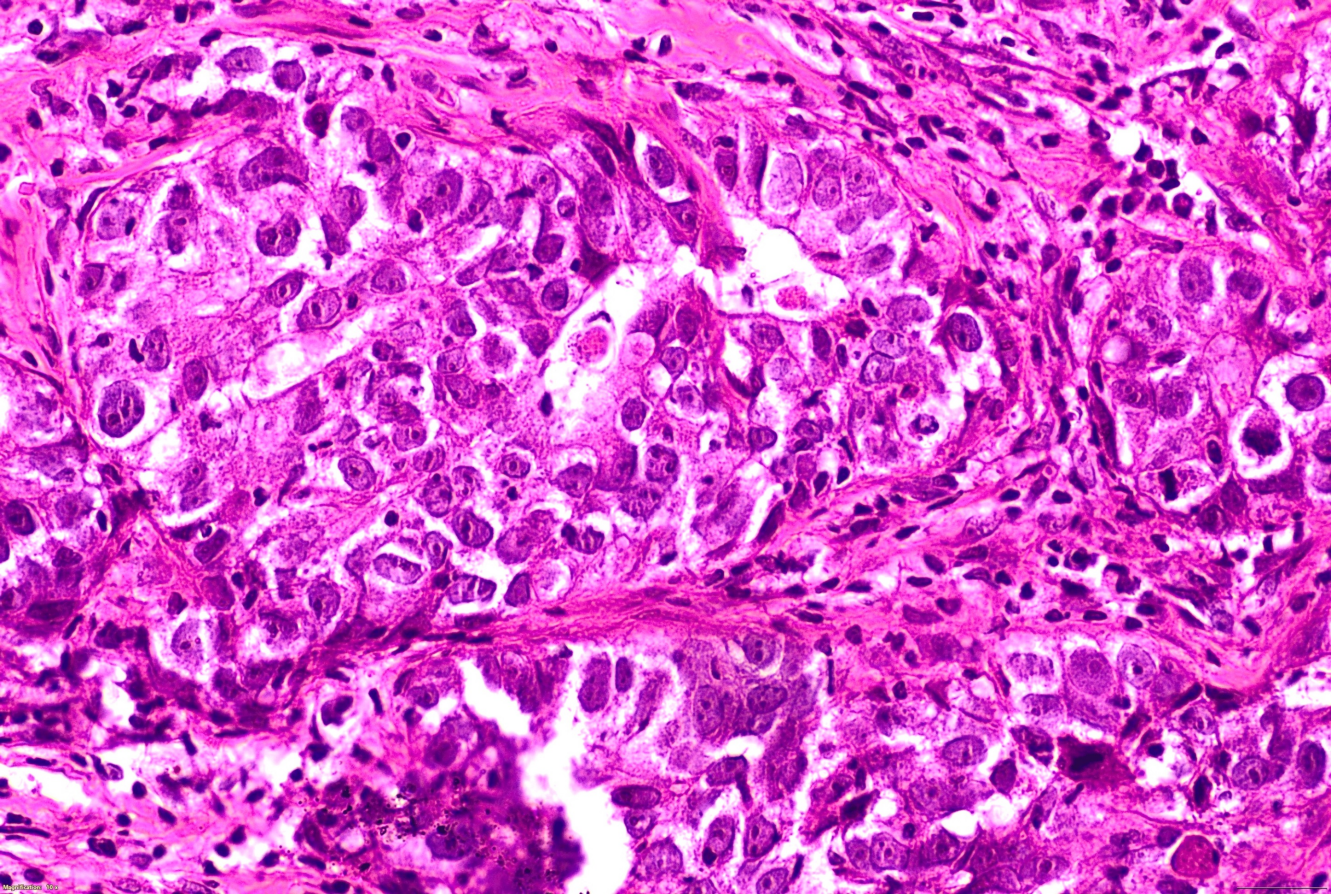
A 400x magnification reveals a high nuclear grade, marked nuclear pleomorphism, and prominent nucleoli.

**Figure 10:**
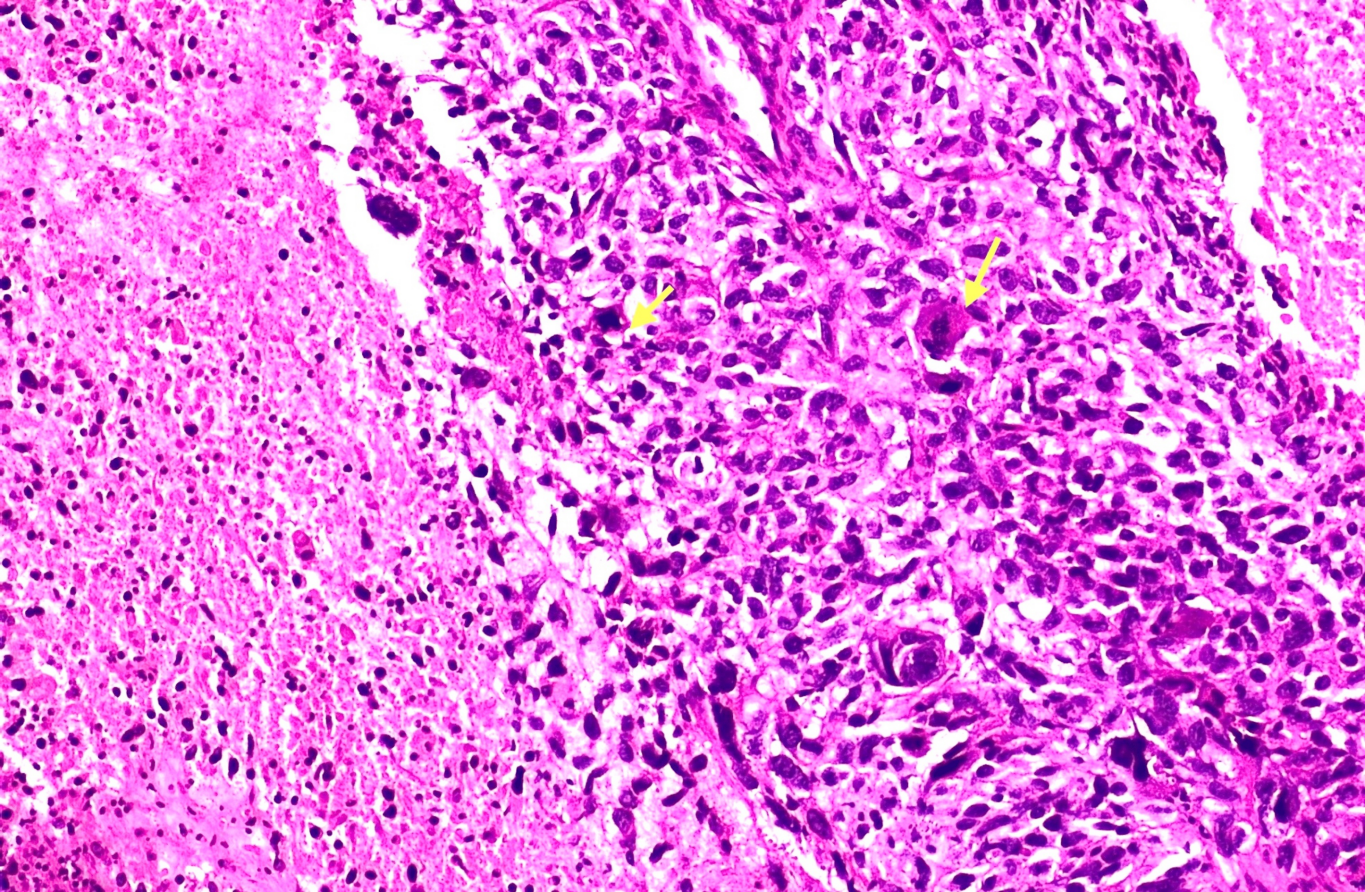
Tumour cells are exhibiting atypical mitosis(indicated by yellow arrowheads), with adjacent areas of necrosis (100x magnification).

All 45 cases included in this study met the WHO criteria for being diagnosed as medullary carcinoma. Forty-two cases (93%) showed a syncytial growth pattern of at least 75% of the tumour volume (Figure 8). Almost 39 (86%) cases demonstrated a high nuclear grade (3), characterised by marked nuclear pleomorphism (Figure 9). The mean mitotic count was 21 per 10 high-power fields (HPFs), indicating a high proliferative activity (Figure 10). A prominent lymphoplasmacytic infiltrate was seen in all tumours, of which 14 (31.1%) cases showed a dense infiltrate, 10 (22.2%) cases showed a moderate infiltrate, and 21 (46.7%) cases demonstrated a mild lymphoplasmacytic infiltrate (Figure 12). Forty-four cases (97.8%) showed no glandular or tubular differentiation. There was focal necrosis observed in the tumour in 12 cases (26.7%). On microscopy, all cases showed a well-defined pushing margin and no evidence of stromal invasion or desmoplastic response, which is consistent with the findings from imaging and gross examinations. The presence of associated ductal carcinoma in situ is exceedingly uncommon, further supporting the concept of MCB as a specific entity with limited progression from in situ components. The results are tabulated in Table 3.

**Figure 11:**
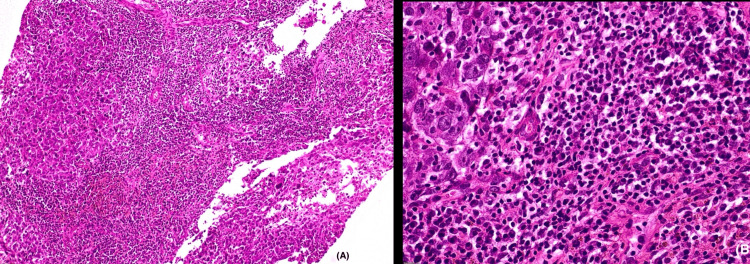
(A) The syncytial growth of tumour cell nests is accompanied by stromal mononuclear lymphoplasmacytic infiltrates (100x). (B) Tumour infiltrating lymphocytes and plasma cells (400x magnification).

**Table 3:** Summary of the histopathological features of medullary carcinoma of the breast (N = 45)

Histopathological findings	Number of tumours (%)
Syncytial growth pattern (>75%)	42 (93%)
Mild lymphoplasmacytic infiltrate	21 (46.7%)
Moderate lymphoplasmacytic infiltrate	10 (22.2%)
Marked lymphoplasmacytic infiltrate	14 (31.1%)
Microscopically circumscribed	45 (100%)
Absence of glandular/tubular differentiation	44 (97.8%)
High-grade nuclear features	39 (86%)

In our cohort, 43 biopsies (95.5%) of the medullary carcinoma exhibited a triple-negative (ER-/PR-/HER2-) immunophenotype and basal-like (EGFR positivity) amplification.

## Discussion

Our comprehensive retrospective study of MCB elaborates on previously established imaging and histopathology characteristics, emphasising their diagnostic relevance. The typical presentation of MCB is a palpable mass, which often occurs in middle-aged women around 50 years old, consistent with the established literature.

The well-circumscribed margins and rounded or oval shape provide a deviation from the typical features of invasive ductal carcinoma [14]. The “benign-like appearance” may lead to an underdiagnosis of the lesion’s malignant potential, which results in the assigning of lower BIRADS categories like 4A/4B, which may delay a definitive diagnosis if a biopsy is not performed. The rarity of calcifications within the MCB, as noted within our study, serves as another distinguishing characteristic, given that calcifications are typically a common indicator associated with other breast cancer subtypes. This feature highlights the necessity of not solely relying on the absence of suspicious calcification to exclude malignancy in any well-defined masses.

Sonography adds further complexity to the diagnostic challenge. Medullary carcinoma consistently exhibited hypoechoic characteristics, which are typical of any solid mass. However, benign lesions like cysts and fibroadenomas are often associated with the striking presence of posterior acoustic enhancement [13]. This phenomenon is associated with high cellularity, minimal desmoplastic reaction, and occasional necrotic and cystic components in medullary carcinomas, which may facilitate the passage of sound waves with reduced attenuation [16]. The well-defined microlobulated margins also contribute to an ambiguous sonographic appearance that may resemble a benign pathology. Therefore, a solid hypoechoic breast mass exhibiting benign characteristics, such as circumscribed margins and posterior enhancement, warrants a careful evaluation for any malignancy, especially medullary carcinoma, and an appropriate biopsy investigation.

The definitive diagnosis of medullary carcinoma relies on histopathological criteria as outlined in the WHO classification [1]. Our study also demonstrated the classic triad: a syncytial growth pattern, dense lymphoplasmacytic infiltrate, and the absence of glandular or tubular differentiation. The syncytial growth pattern is characterised by cohesive sheets of cells with high nuclear grades, indistinct cell borders, and increased mitosis. The lymphoid infiltrate is predominantly composed of T-lymphocytes, which is thought to be due to the host's immune response and contributes generally to the favourable prognosis of medullary carcinoma [8]. The consistent high grade and mitotic rate, despite having a better prognosis, highlight the aggressive cellular biology of this tumour.

The strong correlation between imaging results and the associated histopathology is essential for clinical practice. The well-circumscribed margins, as seen on imaging, correspond directly to the pushing borders, with minimal desmoplastic reaction microscopically. Likewise, the typical homogenous, hypoechoic appearance on ultrasound, accompanied by the posterior acoustic enhancement, reflects the cellular composition with a lack of fibrosis on histology. The higher incidence of a triple-negative immunophenotype in 95% of the cases further emphasises the association of medullary carcinoma with basal-like breast carcinoma, a subtype that has more aggressive clinical behaviour and a favourable response to chemotherapy [11,17]. This molecular profile, together with the unique histological and imaging traits, highlights the necessity of identifying medullary carcinoma as a distinct entity that requires specific diagnostic awareness.

Differential diagnoses for breast masses that are well defined on imaging are broad and include benign conditions like fibroadenomas, cysts, phyllodes tumours, and abscesses, along with malignant lesions like mucinous carcinomas, papillary carcinomas, and certain high-grade IMC-NST [18]. The distinctive combination of imaging (well-circumscribed, hypoechoic, posterior enhancement), combined with a palpable mass in relatively younger and middle-aged women, should raise suspicion of medullary carcinoma and warrant a biopsy. Although MRI was not the main emphasis of this retrospective study, earlier studies indicate that MCBs exhibit a rapid contrast enhancement and washout kinetics on MRI, often displaying smooth or lobulated edges, and at times a pseudo-capsule, which contributes to their distinct appearance [19].

Clinical implications

The findings of this study have significant clinical implications, particularly for the radiologists. Radiologists should be vigilant because medullary carcinoma, despite being malignant, can appear as a benign lesion in conventional imaging techniques such as mammography and ultrasound. A strong level of suspicion is crucial when assessing well-defined breast masses, particularly in patients with a palpable lump. For pathologists, understanding these imaging associations can provide an advantage when interpreting biopsies, particularly in complex cases with overlapping characteristics. Ultimately, accurate diagnosis and effective patient management for this distinctive subtype require a multidisciplinary approach that correlates imaging findings with histopathologic findings.

Limitations

The retrospective design of this study exposes it to several limitations. First, as a study conducted at a single institution, the results may not fully generalise to all populations, and potential biases in patient selection or referral processes cannot be entirely ruled out. Second, despite the assessment of imaging by skilled radiologists, the subjective nature of certain BI-RADS results in variability between observers, even when they reach a consensus. Third, the analysis did not investigate the contribution of other advanced imaging techniques, such as MRI, which could offer further diagnostic insights. Finally, clinical outcomes (such as recurrence and survival rates) were outside the scope of this study, which mainly focused on correlating imaging and pathology. The relatively small sample size for a rare condition like MCB may limit the statistical power to identify subtle relationships, which could lead to type II errors. Future studies with larger cohorts, exploring further modalities, might result in examining more intricate statistical relationships.

## Conclusions

MCB typically presents a unique imaging profile, appearing as a well-defined mass on mammography and as a hypoechoic mass with prominent posterior acoustic enhancement on ultrasound. These characteristics can mimic benign lesions. Histologically, it is characterised by a syncytial growth pattern and a dense lymphoplasmacytic infiltrate. The consistent co-occurrence of posterior acoustic enhancement alongside prominent infiltrates is an important correlative finding. Recognising these distinct multimodal features is essential for accurate diagnosis and avoiding misclassification and underdiagnosis of this distinct subtype of breast cancer.
